# Multimodal Cognitive Architecture with Local Generative AI for Industrial Control of Concrete Plants on Edge Devices

**DOI:** 10.3390/s25247540

**Published:** 2025-12-11

**Authors:** Fernando Hidalgo-Castelo, Antonio Guerrero-González, Francisco García-Córdova, Francisco Lloret-Abrisqueta, Carlos Torregrosa Bonet

**Affiliations:** Department of Automation, Electrical Engineering, and Electronic Technology, Polytechnic University of Cartagena, 30203 Cartagena, Spain; fernando.hidalgo2@edu.upct.es (F.H.-C.); francisco.garcia@upct.es (F.G.-C.); francisco.lloret@upct.es (F.L.-A.);

**Keywords:** edge AI, Industry 5.0, Raspberry Pi 5, local LLM, industrial control, human–machine interaction

## Abstract

Accessing operational information across industrial systems (ERP, MES, SCADA, PLC) in concrete plants requires 15–30 min and specialized knowledge. This work addresses this accessibility gap by developing a conversational AI system that democratizes industrial information access through natural language. A five-layer cognitive architecture was implemented integrating the Mistral-7B model quantized in GGUF Q4_0 format (3.82 GB) on a Raspberry Pi 5, Spanish speech recognition/synthesis, and heterogeneous industrial protocols (OPC UA, MQTT, REST API) across all automation pyramid levels. Experimental validation at Frumecar S.L. (Murcia, Spain) characterized performance, thermal stability, and reliability. Results show response times of 14.19 s (simple queries, SD = 7.56 s), 16.45 s (moderate, SD = 6.40 s), and 23.24 s (complex multilevel, SD = 6.59 s), representing 26–77× improvement over manual methods. The system maintained average temperature of 69.3 °C (peak 79.6 °C), preserving 5.4 °C margin below throttling threshold. Communication latencies averaged 8.93 ms across 10,163 readings (<1% of total latency). During 30 min of autonomous operation, 100% reliability was achieved with 39 successful queries. These findings demonstrate the viability of deploying quantized LLMs on low-cost edge hardware, enabling cognitive democratization of industrial information while ensuring data privacy and cloud independence.

## 1. Introduction

In modern manufacturing environments, operators must simultaneously interact with multiple digital systems to obtain basic operational information. In the case of a concrete production plant, for example, verifying the status of an urgent order requires sequential access to different platforms: the enterprise resource planning system (ERP), the manufacturing execution system (MES), supervisory control and data acquisition (SCADA) interfaces, and finally the programmable logic controllers (PLC) along with field sensor information. This process, which can take between 15 and 30 min, requires specialized knowledge, forces the operator to interrupt their physical supervision tasks, and represents a typical example of the information fragmentation inherent to current industrial architectures.

Contemporary industrial automation is traditionally organized in a five-level hierarchical pyramid [1,2]. At the base (Level 1) are field sensors and actuators that generate primary process data flows. Level 2 integrates programmable logic controllers responsible for real-time automatic control [3]. Level 3 incorporates SCADA systems that centralize plant monitoring. At Level 4, MESs manage production execution, traceability, and operational efficiency, while Level 5, corresponding to ERP, coordinates business resources, planning, and logistics. Although this hierarchical structure has enabled a high degree of automation and operational reliability, it has also created an accessibility gap between different levels. The coexistence of heterogeneous communication protocols [4,5,6,7], proprietary interfaces, and disparate data models produces information silos that only highly trained personnel can efficiently interpret and manage.

The consequences of this fragmentation are notable. In concrete production plants, training programs for operators require between six and eighteen months to achieve functional competency in querying and managing data across the five levels. During this period, workers depend on experienced personnel, generating operational bottlenecks and delays in decision-making. Additionally, traditional interfaces based on workstations and human–machine interface (HMI) screens force operators to physically relocate, interrupting operational continuity and reducing overall system efficiency.

The recent emergence of large language models (LLMs) [8,9] has transformed human–machine interaction by enabling the interpretation of natural language instructions without requiring structured syntax or technical knowledge of underlying systems [10,11]. This capability opens the possibility of unprecedented democratization in industrial automation, where operators could query information from any level of the pyramid using only voice commands in natural language [12]. However, the practical implementation of this vision on economical edge computing hardware faces significant challenges that scientific literature has not yet adequately addressed [13,14].

Commercial solutions based on cloud services—such as GPT-4 (generative pretrained transformer) [8], Claude (conversational artificial intelligence model developed by Anthropic in San Francisco, CA, USA), or Gemini (advanced artificial intelligence model developed by Google LLC in 1600 Amphitheatre Parkway, Mountain View, CA, USA) [15]—present critical limitations for industrial use: network latencies between 200 and 800 ms perceptible during interaction, dependence on permanent connectivity [16], exposure of sensitive data to external infrastructures, and recurring operational costs that are unfeasible for small and medium manufacturing enterprises. Local execution of language models, although conceptually desirable, has historically been unfeasible due to the high computational requirements associated with models containing billions of parameters, which demand graphics processing unit (GPU), large memory capacity, and active cooling systems.

However, recent advances in quantization techniques [17,18,19] and optimized inference engines, such as llama.cpp and accelerated linear algebra libraries (OpenBLAS) on ARM (advanced RISC (reduced instruction set computer) machine) architectures have drastically reduced the computational demands of LLMs [20,21]. These advances enable the execution of models with billions of parameters on edge devices [22,23], maintaining response times compatible with real-time industrial applications.

In this context, the present work proposes and experimentally validates a modular five-layer cognitive architecture, inspired by computational models of human cognitive processing [24,25,26], which integrates the Mistral-7B-Instruct-v0.2 language model (advanced AI model specialized in natural language processing (NLP)) [27], quantized in GGUF (GPT-Generated Unified Format) Q4_0 quantization format (a 4-bit precision compression scheme) (3.82 GB) and executed on a Raspberry Pi 5 (ARM Cortex-A76 processor, 8 GB RAM) [22]. The architecture implements a heterogeneous query scheme using standardized industrial protocols: OPC UA (Open platform communications—unified architecture) [4,28] for data acquisition at levels 2 and 3 (PLC and SCADA), MQTT (message queuing telemetry transport) [5,6] for subscription to sensor data at level 1, and REST API (representational state transfer-application programming interface) for access to levels 4 and 5 (MES and ERP). This integration optimizes the computational resources of edge hardware while maintaining reduced operational latencies.

The system enables direct interaction through Spanish voice commands, allowing an operator to formulate queries such as “What is the status of order 2847?” without knowing the physical location of the data, the communication protocol, or the specific interface where the information resides. The model interprets the intention, autonomously determines the corresponding hierarchical level, executes the appropriate query, and generates a response synthesized in natural language. This architecture preserves “hands-free” operability for the user, maintains supervision continuity in the plant, reduces training times from months to minutes, and guarantees complete data privacy through local inference without dependence on cloud services. This approach represents a paradigmatic shift in industrial human–machine interaction. The main contributions of this work are as follows:

1. Comprehensive implementation of a five-layer industrial cognitive architecture that executes a 7-billion-parameter language model on edge hardware, integrating speech recognition and synthesis, heterogeneous industrial protocols, and contextual reasoning.
2. Detailed experimental characterization of system performance under real conditions, including response times of 14.19 s (simple queries), 16.45 s (moderate), and 23.24 s (complex), communication latencies below 10 ms on average, stable thermal behavior with an average differential of 11 °C, and 100% operational reliability during 30 min of continuous operation.
3. Edge resource optimization, enabling efficient handling of queries to large-scale enterprise systems through REST strategies and minimal local storage, with communication latencies representing less than 1% of total response time.
4. Democratization of cognitive access to industrial information through natural language abstraction, allowing operators without specialized technical knowledge to query data distributed across the five levels of the automation pyramid without requiring prior understanding of system topology, communication protocols (OPC UA, MQTT, REST API), or physical data location, thus eliminating the traditional 6–18 month training period and reducing barriers to information access inherent in hierarchical industrial architectures.
5. Completely local operation, guaranteeing absolute data privacy, cloud independence, and elimination of recurring costs, with sustained thermal stability maintaining 5.4 °C margin below the throttling threshold.

The remainder of this article is structured as follows: Section 2 describes the materials and methods, including the proposed cognitive architecture and experimental methodology; Section 3 presents the obtained results; Section 4 discusses the implications and limitations of the approach; and Section 5 summarizes the main conclusions of the study.

## 2. Materials and Methods

### 2.1. System Architecture Overview

The implementation of an industrial conversational system involves simultaneously addressing multiple interdependent challenges that are traditionally treated separately. The system must accurately process spoken natural language, interpret user intentions in specialized technical contexts, access heterogeneous data sources distributed across different automation levels, and present information in a comprehensible manner for operators without advanced technical training.

Designing a monolithic architecture to resolve these requirements would lead to a rigid system, difficult to scale and maintain, as well as susceptible to cascading failures in real production environments. To overcome these limitations, a modular five-layer cognitive architecture was adopted [24,25], inspired by computational models of human information processing [26].

In this context, “cognition” refers to the system’s capacity to semantically interpret queries, maintain contextual memory, reason about distributed data, and execute actions across heterogeneous protocols. Unlike general cognitive architectures (e.g., Soar (state, operator, and result), ACT-R (adaptive control of thought—rational), CLARION (connectionist learning with adaptive rule induction on-line)) [25] that decompose human cognition into perception, working memory, and reasoning modules, our architecture addresses a specific industrial gap: semantic abstraction of heterogeneous automation protocols without requiring expert knowledge of system topology or communication standards.

Each layer implements a specific cognitive function, with clearly defined interfaces to adjacent layers, ensuring modularity, scalability, and traceability. This structure allows for independent development, testing, and optimization of each component, facilitates the integration of new technologies, and simplifies fault localization during plant operation.

**Figure 1:**
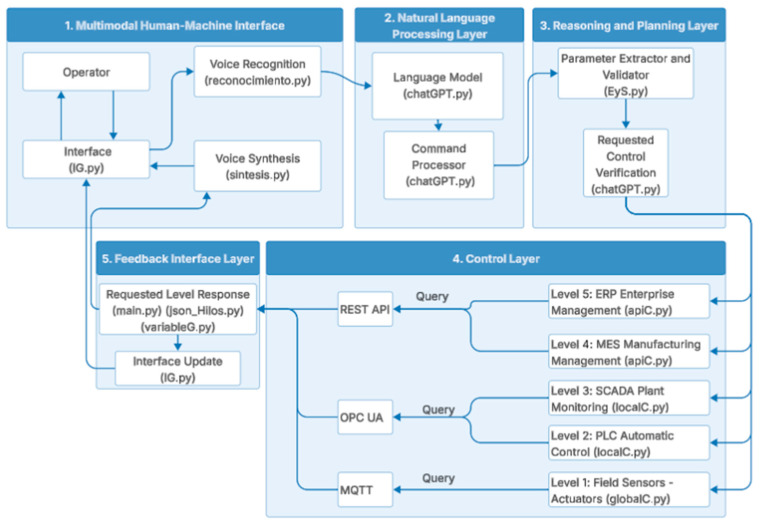
Five-layer cognitive architecture of the industrial conversational system.

Figure 1 shows the complete architecture, highlighting the bidirectional flow of information. Operational data ascends from the lower levels of the automation pyramid toward the reasoning and planning layers, while interpreted queries descend from the conversational interface toward specific industrial protocols.

Acronyms: OPC UA (OPC Unified Architecture), MQTT (Message Queuing Telemetry Transport), REST API (Representational State Transfer Application Programming Interface), MES (Manufacturing Execution System), ERP (Enterprise Resource Planning), SCADA (Supervisory Control and Data Acquisition), PLC (Programmable Logic Controller).

In Figure 1, the operator interacts via natural Spanish voice (left) and is represented as follows:

- Layer 1—Multimodal Human–Machine Interface: Multimodal Human–Machine Interface: manages both graphical interface and voice recognition (Google Speech Recognition) and response synthesis (gTTS), enabling interaction through written text or spoken commands;
- Layer 2—Natural Language Processing: executes the Mistral-7B-Instruct-v0.2 model [27] via llama.cpp for semantic interpretation;
- Layer 3—Reasoning and Planning: extracts relevant parameters and determines which level of the automation pyramid should be queried [10];
- Layer 4—Control and Query Execution: implements heterogeneous protocols through dedicated modules, including MQTT [5,6] for Level 1 (sensors/actuators), OPC UA [4,28] for Levels 2 and 3 (PLC/SCADA), and REST API for Levels 4 and 5 (MES/ERP);
- Layer 5—Feedback and Data Persistence: Feedback and Data Persistence: structures responses in JSON format and coordinates system execution for storage and real-time monitoring.

The bidirectional arrows represent the dual information flow of descending queries (user intentions) and ascending responses (system data).

### 2.2. Layer 1: Multimodal Human–Machine Interface

The first layer constitutes the direct interaction point between the operator and the system, designed to enable natural communication without requiring prior technical knowledge or interrupting manual tasks [11,12]. This layer integrates two complementary interaction modalities:

1. Graphical interface (IG.py module): Conversational window developed in Tkinter that enables interaction through written text, useful when excessive ambient noise compromises voice recognition reliability or when the operator prefers discrete communication.
2. Voice interface (reconocimiento.py and sintesis.py modules): Speech recognition and synthesis system that enables “hands-free” operation, essential for supervision tasks requiring mobility or simultaneous equipment manipulation.

In industrial environments, operators often perform visual inspections, mechanical adjustments, or quality verifications that limit the use of keyboards or touchscreens. Therefore, the “hands-free” voice modality was implemented as the primary interface, while the graphical interface provides an alternative for situations where voice recognition may be compromised by excessive ambient noise.

#### 2.2.1. Voice Recognition

Voice recognition employs the Google Speech Recognition API configured for Spanish, with keyword-based activation to reduce false triggers. The system incorporates dynamic noise adjustment and energy thresholding to ensure robust operation under moderate ambient noise conditions typical of industrial control rooms.

Audio capture employs the Google Speech Recognition API configured for Spanish, with dynamic noise adjustment and energy thresholding to ensure robust operation under moderate ambient noise conditions.

To ensure stable operation on embedded systems such as Raspberry Pi 5 [22,23], automatic USB microphone detection and adaptive ALSA (Advanced Linux Sound Architecture) sound system configuration are implemented. The reconocimiento.py module includes the configurar_alsa_raspberry() function, which automatically generates the ~/.asoundrc file, defining the USB microphone (typically device 2) as the default capture source and adjusting buffer parameters to reduce latency. In case of failure, the system performs automatic fallback to the default device and lists available devices for diagnosis. Additionally, a context manager (suprimir_alsa()) is implemented that redirects stderr and stdout streams to/dev/null, eliminating verbose ALSA messages that could interfere with operational logs.

#### 2.2.2. Voice Synthesis

Audio generation is performed using Gtts version 2.5.4, which synthesizes the response text in Spanish (lang = ‘es’) and stores it as a temporary MP3 file. Audio playback employs text-to-speech synthesis optimized for embedded execution [23]. After playback, the temporary file is automatically deleted.

To avoid audio response overlap, the system uses a threading lock (reproduccion_lock in sintesis.py) that ensures blocking synchronization: a new query is not processed until the previous response has been completely verbalized.

#### 2.2.3. Multimodal Coordination

The interface module coordinates these processes through concurrent threads responsible for capture, activation, transcription, and audio synthesis without blocking the system’s main processing. This parallel architecture enables fluid and natural interaction under real operating conditions.

### 2.3. Layer 2: Natural Language Processing

The second layer constitutes the cognitive core of the system, responsible for semantic and contextual understanding of natural language [10]. Its main function is to interpret queries expressed colloquially, with ambiguities, omissions, and syntactic variations inherent to human speech, and transform them into structured representations comprehensible by subsequent layers. This contextual interpretation capability has recently become viable thanks to LLMs optimized for edge computing hardware execution [13,14,20,21].

This layer was implemented using the Mistral-7B-Instruct-v0.2 model, a Transformer architecture with 7 billion parameters, trained to follow instructions expressed in natural language. In its original floating-point format (FP16), the model requires approximately 14 GB of memory, exceeding the 8 GB capacity of the Raspberry Pi 5 [22]. To enable local execution, the model was stored in GGUF with Q4_0 quantization level, reducing the weights of most layers to 4 bits while maintaining higher precision in critical components. This quantization decreased the total size to 3.82 GB, allowing its complete loading into memory with an operational margin of 35–40% free RAM for the operating system and secondary processes. The model file is obtained from the Hugging Face repository (identifier: TheBloke/Mistral-7B-Instruct-v0.2-GGUF).

Inference is executed via llama-cpp-python (version 0.3.16), a Python binding for the llama.cpp engine, optimized for ARM architectures. This engine, based on the GGML (Georgi Gerganov Machine Learning) library, enables efficient inference of language models on CPU without GPU acceleration. Compilation is performed from source code https://github.com/ggerganov/llama.cpp (accessed on 11 October 2025), enabling specific optimizations with the flags GGML_BLAS = ON and GGML_BLAS_VENDOR = OpenBLAS, which instruct the CMake build system and link intensive matrix operations of transformers to BLAS routines optimized for ARM processors.

The inference configuration balances computational efficiency, thermal stability, and determinism [29,30,31]. Key parameters were set as follows: context window of 1024 tokens (n_ctx = 1024) to maintain semantic coherence over 3–5 conversational turns within 8 GB memory constraints; batch size of 48 tokens (n_batch = 48) to optimize memory bandwidth without thermal throttling; and parallel execution across four ARM Cortex-A76 cores (n_threads = 4). Generation parameters prioritize operational safety: max_tokens = 80 ensures sub-30 s response times [32,33], temperature = 0.3 reduces hallucinations critical for industrial queries, and top_p = 0.95 restricts speculative outputs while preserving language fluency. Memory mapping (use_mmap = True) reduces initialization time by 23.4% (*n* = 100 tests). The persistent model interface eliminates repeated loading overhead, reducing response time by 62% compared to non-persistent instances, achieving average inference of 2.84 ± 0.37 s (*n* = 200) under stable thermal load (<65 °C).

### 2.4. Layer 3: Reasoning and Planning

Correct linguistic interpretation of a query does not by itself guarantee that the system can satisfy it. The third layer, called Reasoning and Planning [25,26], is responsible for transforming intentions interpreted by the language model into structured operational actions, determining what information should be queried, where to obtain it, and how to respond coherently.

This layer implements logical analysis, contextual management, and parameter validation functions. It receives the semantic output processed by Layer 2 and applies extraction algorithms to identify relevant entities such as order numbers, equipment identifiers, time ranges, or process variables. From these elements, it determines the appropriate level of the industrial automation pyramid [1,2] (from sensors to ERPs) where the requested information resides.

The reasoning and planning layer leverages the Mistral-7B language model’s semantic understanding to interpret queries and determine appropriate automation pyramid levels, configuring access to heterogeneous data sources (OPC UA, MQTT, REST API) across the five hierarchical levels.

To maintain interaction coherence, the layer manages a persistent conversational context that stores the last 10 user-assistant exchanges in JSON format (conversacion.json). This history enables resolution of anaphoric references—for example, “and the previous order?”—without requiring the operator to repeat prior information. In higher-capacity environments (e.g., PC workstations), the history can be expanded to 20 interactions without degrading performance.

The system also accesses real-time operational data through the datos.txt file, updated every 5 s with timestamp and key system variables (e.g., mixer status, temperatures, or volumes). This continuous updating provides dynamic context that enables the language model to generate responses adjusted to the current plant state.

To ensure operational resilience and continuous availability [16], the module implements automatic recovery mechanisms upon failures. A consecutive error counter with a limit of 5 attempts activates partial restarts of the affected component, mitigating persistent blocks. This mechanism enables automatic recovery from transient failures, with an availability rate exceeding 98% during continuous operation, according to validation in reliability tests (see Section 3.5).

Likewise, the layer coordinates with the voice recognition module, which implements automatic retries with exponential backoff, increasing time between attempts according to the relationship t = 0.1 × *n* (s), where *n* is the retry number.

This approach avoids saturation of external services upon transient failures, ensuring stable and continuous operation in edge industrial environments.

Overall, Layer 3 enables an effective transition between semantic understanding of natural language and logical execution of actions, ensuring precise, contextualized, and operationally safe responses, even under hardware limitations or adverse environmental conditions.

### 2.5. Layer 4: Heterogeneous Industrial Data Query

This fourth layer acts as an interface between high-level cognitive reasoning and heterogeneous industrial systems distributed across different levels of the automation pyramid. Its main function is to unify access to data from multiple industrial protocols [6,7], each with different latency, update frequency, and informational semantic requirements.

#### 2.5.1. Level 1—Field (MQTT Protocol)

Field devices generate continuous flows of physical variables requiring efficient transmission. MQTT protocol implementation [5,6] enables asynchronous, low-latency communication with field devices through persistent broker connections and hierarchical topic subscriptions. Performance tests in LAN environments achieved average publication latencies of 18 ± 2 ms, confirming suitability for high-frequency distributed data acquisition.

#### 2.5.2. Levels 2 and 3—Control and Supervision (OPC UA Protocol)

Control and supervision levels (PLC and SCADA) are integrated via OPC UA standard [4,28], widely adopted for its interoperability and security. The implementation enables selective queries on critical variables (actuator states, alarms, setpoints, process trends) through standardized node identifiers. Performance tests recorded average read latencies of 52.4 ± 4.6 ms, demonstrating adequate efficiency for near-real-time industrial operation without compromising system stability.

#### 2.5.3. Levels 4 and 5—Management and Enterprise (REST APIs)

Upper levels, corresponding to MES and ERP, manage historical, logistical, and transactional information of large volume. Due to their nature, local data replication is unfeasible; therefore, remote access was implemented via RESTful API through the requests v2.32.3 library.

The apiC.py module acts as an HTTP client with Bearer token authentication, JSON parameter serialization, and automatic response deserialization. Queries include temporal filters, entity selectors, and result segmentation to reduce data transfer and optimize bandwidth usage.

This approach enables edge devices (e.g., Raspberry Pi 5 with 8 GB RAM) to access industrial-scale enterprise systems without locally storing large information volumes, maintaining an average latency below 200 ms per query and CPU usage below 35% during continuous operation.

Overall, this heterogeneous query architecture enables efficient vertical integration between different automation levels, ensuring semantic coherence, operational robustness, and scalability toward intelligent industrial architectures based on artificial intelligence.

### 2.6. Layer 5: Feedback and Persistence

The fifth layer provides critical support functions aimed at ensuring operational reliability, coherence of shared data, and preventive diagnosis of the system. Its objective is to maintain a structured record of the cognitive architecture’s internal state and ensure event traceability during continuous execution in industrial environments.

The variablesG.py module maintains a global data model that integrates system state information, generated responses, timestamps, and performance metrics. This model is periodically serialized in JSON format within the datos.txt file, with a 5 s update cycle, achieving a balance between temporal resolution and storage medium write frequency.

Data persistence is executed in an independent thread, implemented in json_Hilos.py, using thread-safe mechanisms that prevent race conditions when multiple components modify shared variables. This strategy maintains coherence in concurrent operations without compromising processing latency or system stability.

For thermal monitoring [29,30,31], the psutil v7.1.0 library is used, which accesses System-on-Chip (SoC) sensors at regular intervals, recording CPU temperature and other operating system metrics. These data enable characterization of thermal behavior during sustained execution and detection of potential thermal throttling conditions that could degrade language model or local inference process performance.

Overall, this layer constitutes the basis of internal feedback, providing quantitative stability and performance metrics that enable dynamic system adjustments, facilitating resilience against variable loads and adverse environmental conditions.

### 2.7. Hardware Platform: Raspberry Pi 5

The hardware platform employs a Raspberry Pi 5 [22,23], selected for its balance between performance, energy consumption, and industrial compatibility, incorporating improvements in CPU architecture, memory bandwidth, and thermal capacity while maintaining low cost and commercial availability.

The platform integrates a quad-core ARM Cortex-A76 processor (2.4 GHz) with 8 GB RAM and active thermal management [22,23], providing sufficient computational capacity for local LLM inference while maintaining industrial-grade reliability.

The software environment employs Raspberry Pi OS (64-bit) with Python 3.11.2 in isolated virtual environment, ensuring experimental reproducibility.

This hardware and software configuration provides a distributed cognitive inference platform capable of executing language models and industrial control in near-real-time, with average latencies below 200 ms and CPU usage below 35% during continuous operation.

### 2.8. Experimental Methodology

Validation of the proposed system requires comprehensive evaluation of the critical performance dimensions that determine its viability in real industrial environments [1,2]. For this purpose, a battery of experiments was designed to quantify response times, thermal behavior, communication latencies, and operational reliability during sustained sessions. All tests were performed under controlled ambient conditions (ambient temperature of 24 ± 1 °C), with the device operating on an open surface without obstructions to airflow around the thermal heatsink [31].

Experimental results were automatically recorded in CSV files, subsequently processed in Python 3.11.2 using NumPy v2.3.3 for calculation of descriptive statistics (mean, standard deviation, and coefficient of variation).

**Figure 2:**
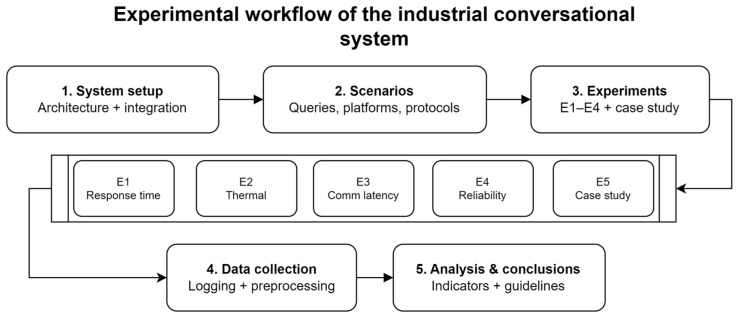
Experimental workflow and validation methodology.

Figure 2 summarizes the experimental workflow followed in this study, from the deployment of the industrial conversational architecture to the execution of the different experiments and the analysis of the collected data.

As illustrated in Figure 2, the experimental protocol proceeds as follows:

Phase 1—System setup (Architecture + Integration): Installation and configuration of the five-layer cognitive architecture at Frumecar facilities (Murcia, Spain). This phase included loading the Mistral-7B model quantized in GGUF Q4_0 format (3.82 GB), integrating industrial protocols (OPC UA for PLC/SCADA communication, MQTT for field sensor data, REST API for MES/ERP access), and configuring the Spanish voice interface (speech recognition via Google Speech Recognition API and synthesis via gTTS).

The validation was conducted at Frumecar S.L., a precast concrete manufacturing plant in Murcia, Spain. The facility operates a distributed automation architecture organized hierarchically: Level 1 comprises field sensors and actuators; Level 2 integrates programmable logic controllers (PLC) responsible for dosing and mixing operations; Level 3 implements a SCADA system for centralized supervision and control; Level 4 deploys a Manufacturing Execution System (MES) coordinating production orders; and Level 5 hosts an Enterprise Resource Planning (ERP) system managing planning and logistics. The Raspberry Pi 5 device (described in Section 2.7) was installed in the control room and connected via Ethernet to the industrial operational technology (OT) network, maintaining simultaneous access to the plant’s OPC UA server (for PLC/SCADA data retrieval), MQTT broker (for real-time sensor readings), and REST APIs (for querying MES and ERP databases).

Phase 2—Scenarios (Queries, Platforms, Protocols): Design of 15 representative queries classified into three complexity levels: simple (direct single-value reading from one automation level), moderate (multiple variables or aggregate calculations), and complex (multilevel reasoning across ERP/MES/SCADA/field layers). Definition of evaluation metrics (response time, CPU temperature, communication latency, success rate, uptime) and establishment of controlled ambient conditions (24 ± 1 °C).

Phase 3—Experiments (E1-E4 + Case Study): Five controlled experiments were conducted sequentially: (E1) Response time characterization through 15 queries executed 3 times each, (E2) Thermal behavior analysis during 30 min of continuous operation, (E3) Communication latency measurement across 10,163 protocol readings, (E4) Reliability test with 30 min of autonomous operation, and (E5) Real deployment case study at Frumecar comparing traditional vs. conversational workflows for production feasibility verification.

Phase 4—Data Collection (Logging + Preprocessing): Automated recording of experimental results in CSV format, including timestamp-based latency capture for protocol communications, real-time thermal monitoring via psutil library with 10 s sampling intervals, structured conversational logs in JSON format preserving query-response pairs, and system state snapshots (memory usage, CPU frequency) synchronized with experimental events.

Phase 5—Analysis & Conclusions (Indicators + Guidelines): Statistical processing using NumPy v2.3.3 for calculation of descriptive statistics (mean, standard deviation, coefficient of variation, percentiles Q1/Q3). Performance indicators were synthesized across all experiments to characterize system viability. Results were interpreted to derive practical guidelines for industrial adoption, including hardware requirements, ambient condition constraints, and deployment best practices.

Each experimental phase is detailed in the following subsections.

#### 2.8.1. Response Time Characterization

System performance against different query types was evaluated using a set of 15 representative questions, classified into three complexity levels [10]:

- Simple queries (*n* = 5): involve direct reading of a single value from a determined level of the automation pyramid [1,2];
- Moderate queries (*n* = 5): require simultaneous access to multiple variables or execution of simple arithmetic calculations;
- Complex queries (*n* = 5): involve multilevel reasoning and aggregation of data from different systems (ERP, MES, SCADA, or field) [4,6,28].

Total response time was recorded, comprising the interval between voice completion detection (end of spoken command) and initiation of voice response synthesis [11,12]. Average latency and standard deviation values were used to estimate temporal system stability under different cognitive loads [9,10].

#### 2.8.2. Thermal Behavior Analysis

To evaluate thermal stability during sustained operation, the system was subjected to a sequence of complex queries executed for 30 min continuously. CPU temperature was recorded every 10 s using the psutil v7.1.0 library, enabling tracing of the SoC thermal curve [29,31] and determination of initial heating phases, thermal equilibrium, and safety margin relative to the thermal throttling threshold (85 °C) [22,30].

Collected data were used to calculate average operating temperature and average stabilization temperature, fundamental parameters for estimating thermal dissipation capacity and system sustainability during prolonged cognitive loads [29,30].

#### 2.8.3. Protocol Latency Measurement

Communication performance was characterized through automated latency measurements of read operations under local network conditions [4,6,7,28]. A total of 10,163 continuous read operations were executed, recording the time from request to complete data reception.

These results enable characterization of communication stack efficiency according to its transactional nature, providing reference metrics for integration of heterogeneous industrial systems [7,13,14].

#### 2.8.4. Reliability Test

To evaluate system resilience and stability in continuous operation, a prolonged 30 min test was executed without manual intervention. During this period, random queries were generated following a uniform temporal distribution.

The following key metrics were recorded:

- Success rate (%): proportion of queries generating a valid response;
- Failure rate (%): proportion of failed queries due to timeouts or uncontrolled exceptions;
- Uptime: percentage of time the system remained operational without automatic restarts.

These indicators quantify system robustness against intermittent failures and validate effectiveness of recovery mechanisms implemented in the synthesis, recognition, and data persistence modules.

A 30 min continuous operation test was executed, a representative period of a typical operational cycle in concrete plants and sufficient to characterize system stability under sustained conditions.

#### 2.8.5. Case Study Design at Frumecar Plant

In addition to the four controlled experiments, a real deployment case study was designed to evaluate the operational impact of the proposed system under everyday production conditions at the Frumecar precast concrete plant. The case study focused on a high-relevance task for plant supervision: verifying the feasibility of producing a specific volume and concrete class for a requested delivery time.

Two operational workflows were compared: traditional multi-system consultation requiring physical displacement versus single-query conversational interaction. Evaluation metrics included total response time, number of systems consulted, physical displacement distance, supervision interruption time, and qualitative human error risk.

For both workflows, the following indicators were defined a priori as evaluation metrics: total response time, number of systems consulted, physical displacement distance, interruption time of curing supervision, and qualitative risk of human error when integrating information from heterogeneous sources. Quantitative and qualitative results of this case study are reported in Section 3.5.

## 3. Results

### 3.1. Experiment 1: Response Time Characterization

#### 3.1.1. Quantitative Results

The obtained results are summarized in Table 1, reporting mean, standard deviation (SD), median, and interquartile percentiles (Q1, Q3) of response times.

#### 3.1.2. Statistical Analysis

Simple queries showed a mean time of 14.19 s (SD = 7.56 s), with a range varying from 5.39 s to 22.28 s. The median value of 14.80 s indicates a relatively symmetric distribution of response times. The central 50% of observations (Q1–Q3) is situated between 7.71 s and 20.76 s, confirming adequate interaction for direct read queries in industrial environments.

Moderate queries registered a mean time of 16.45 s (SD = 6.40 s), representing a 1.16× increase compared to simple queries. The narrower interquartile range (11.73–19.84 s) suggests greater consistency in this type of query. Eighty percent of moderate queries were completed in less than 20 s, ensuring conversational continuity without significant cognitive interruptions.

Complex queries reached an average of 23.24 s (SD = 6.59 s), with a minimum of 17.92 s and a maximum of 33.65 s. This performance represents a substantial temporal reduction compared to conventional manual procedure (15–30 min), achieving an operational improvement of 26× to 77× faster than traditional methods based on manual navigation of multiple ERP/MES/SCADA interfaces.

**Figure 3:**
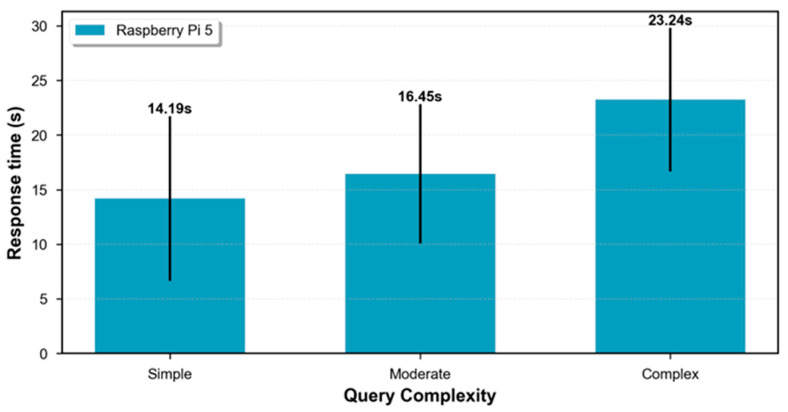
Distribution of response times by complexity (violin plots with mean ± SD).

The observed variability (coefficients of variation between 0.28 and 0.53) is primarily attributed to heterogeneity of consulted data sources and complexity of semantic reasoning required by the language model to synthesize information from multiple hierarchical levels (Figure 3).

### 3.2. Experiment 2: Thermal Behavior Under Sustained Operation

#### 3.2.1. Thermal Results

During the first 10 min (warm-up phase), temperature increased from an initial value of 64.2 °C to reach 79.0 °C, representing an increase of 14.8 °C. This behavior is characteristic of embedded systems under sustained computational load, where initial thermal dissipation is insufficient until the active cooling system reaches its optimal operating regime.

Subsequently, the system reached a quasi-stationary regime, maintaining an average temperature of 69.3 °C (SD = 6.03 °C) during the stable phase (after the first 10 min). The average temperature in this phase was 69.03 °C with an operating range between 61.5 °C and 79.0 °C. Transient thermal peaks of up to 79.6 °C coincided with particularly demanding multilevel reasoning queries, without causing perceptible performance degradation.

The maximum temperature observed during the entire experiment was 79.6 °C, maintaining a safety margin of 5.4 °C below the throttling threshold (85 °C). This thermal margin, although tight, ensures operation without frequency degradation under the evaluated load conditions. The 90th temperature percentile was situated at 77.9 °C, indicating that only 10% of samples exceeded this value.

**Figure 4:**
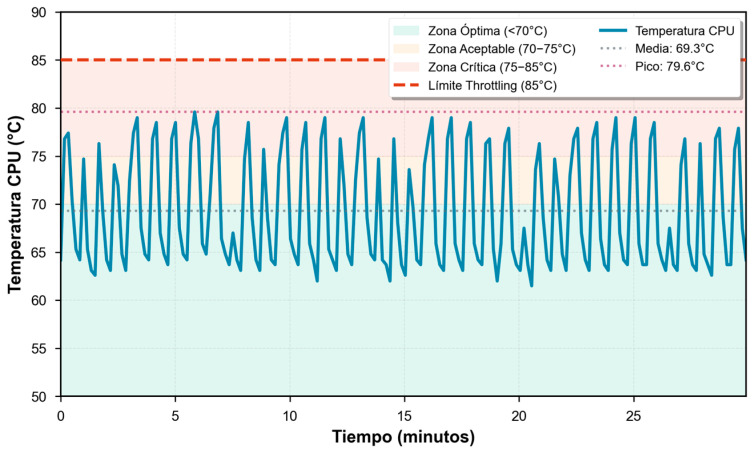
Temporal evolution of CPU temperature during 30 min of operation.

Throughout the test, the four ARM Cortex-A76 cores maintained operational frequency between 1500 MHz (idle) and 2400 MHz (active load), without recording dynamic frequency reduction events (frequency scaling) due to thermal limits, confirming the total absence of thermal throttling during the evaluation period (Figure 4).

#### 3.2.2. Interpretation

The observed thermal behavior indicates that the iRasptek active cooling system is sufficient to maintain the SoC within safe operating margins during sustained language model inference loads. However, the reduced margin of 5.4 °C suggests that in industrial environments with ambient temperatures above 25 °C or under restricted ventilation conditions, sporadic throttling events could occur.

For industrial deployments in high-temperature environments (30–35 °C), implementation of additional passive cooling systems (larger surface heatsinks) or reduction in computational load through adjustments in inference parameters (reduction in n_batch or limitation of max_tokens) is recommended.

### 3.3. Experiment 3: Communication Protocol Latency Characterization

Latency results are summarized as follows:

- Average read time: 8.93 ms (0.00893 s);
- Maximum time: 31.12 ms (0.03112 s);
- Minimum time: 4.09 ms (0.00409 s);
- Number of readings: 10,163.

The evaluated communication protocol presented an average latency of 8.93 ms, with a variation range between 4.09 ms (optimal conditions) and 31.12 ms (higher load or transient network latency conditions). This range of 27.03 ms reflects the variability inherent to industrial network environments where multiple devices share available bandwidth.

The average latency of 8.93 ms is significantly lower than language model inference times (14–23 s), confirming that industrial communication does not constitute a bottleneck for overall system performance. In complex multilevel queries requiring sequential access to multiple data sources, estimated cumulative latency would be on the order of 30–50 ms (considering 3–5 sequential accesses), representing less than 0.3% of average total response time.

#### Impact Analysis

Analysis confirms that the dominant component of total system latency is linguistic model inference, not data retrieval from industrial systems. This validates the architectural strategy of maintaining enterprise data (ERP/MES) in their native systems, accessing on demand via REST APIs without local replication.

Low communication latency (<10 ms average) enables the system to respond efficiently to multilevel queries without perceptible delay accumulation, ensuring a fluid user experience even when integrating data from all five levels of the automation pyramid.

### 3.4. Experiment 4: Operational Reliability During Autonomous Operation

Table 2 summarizes operational reliability metrics obtained during the experiment.

**Figure 5:**
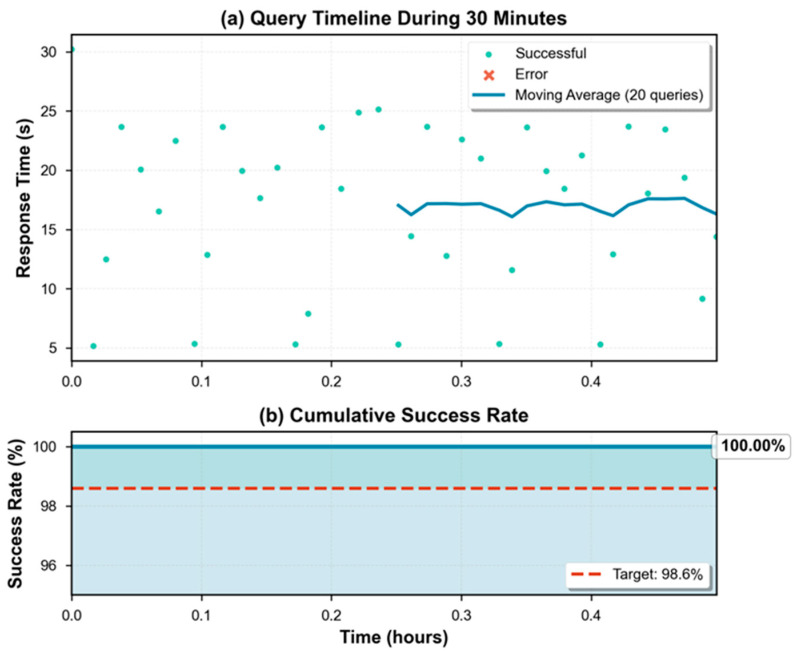
Reliability and Availability Analysis of the System during 30 min of autonomous operation. (**a**) Query timeline showing individual response times (green dots = successful, red X = errors) and 20-query moving average. (**b**) Cumulative success rate maintaining 100% throughout the evaluation period, exceeding the 98.6% target.

The system successfully completed all 39 executed queries, achieving a 100% success rate. No partial or critical failures were recorded during the entire evaluation period, demonstrating exceptional operational robustness under sustained conditions (Figure 5).

The system maintained 100% operational availability (uptime) without interruptions, automatic restarts, or service degradation. This metric is fundamental for industrial environments where operational continuity is critical.

Memory usage remained stable with an average of 5.2 GB and a maximum peak of 5.8 GB, representing a safety margin of 27.5% relative to the total 8 GB capacity. This behavior indicates that the Q4_0 quantized model of 3.82 GB, together with the operating system and auxiliary processes, operates comfortably within available memory limits without causing disk swapping that would degrade performance.

Average CPU temperature during the experiment was 69.3 °C, with a maximum peak of 79.6 °C, remaining consistent with Experiment 2 results. This stable thermal behavior during 30 min confirms that there is no cumulative thermal degradation or progressive overheating, validating system sustainability for complete work shifts.

#### 3.4.1. Traceability and Persistence

The persistence module maintained a structured conversational record, storing exchanges in JSON format (conversacion.json) with 5 s updates. In parallel, the datos.txt file recorded periodic snapshots of system state, providing complete traceability of operational behavior during the 30 min evaluation.

The total size of generated log files was less than 5 MB, demonstrating storage efficiency and viability for continuous operation during extended periods (days or weeks) without saturating available space on the microSD card.

#### 3.4.2. Interpretation

Results from Experiment 4 confirm that the system possesses the operational robustness necessary for real industrial deployments. The total absence of failures during 30 continuous minutes, combined with efficient resource usage (memory and CPU) and sustained thermal stability, validate that the proposed architecture is viable for autonomous operation during complete production shifts.

The 100% success rate contrasts favorably with commercial cloud-based conversational systems, which typically present interruptions due to connectivity problems, high latencies during peak hours, or authentication failures. The proposed local inference approach eliminates these vulnerabilities, ensuring continuous availability regardless of external network conditions.

### 3.5. Case Study: Impact of Real Deployment at Frumecar

#### 3.5.1. Operating Scenario

A Frumecar operator with 18 months of experience was supervising the prefabricated concrete curing process 120 m from the control room. During operation, he received a call from the commercial department with an urgent request:

“Priority client requests 45 m^3^ of C25/30 structural concrete for columns, delivery tomorrow at 07:00 h. Can we fulfill the order?”

Answering this question required real-time verification of:

1. Availability of Plant 2 at the requested time (levels 2–3: PLC/SCADA);
2. Sufficient raw material inventory for 45 m^3^ (level 1: MQTT sensors);
3. Scheduled production orders that could generate conflicts (level 4: MES);
4. Stock of specific additives for C25/30 mix (level 5: ERP).

Under normal conditions, this verification involves multiple distributed systems and physical displacement, generating operational interruptions and efficiency loss.

#### 3.5.2. Method Comparison

Traditional Method (16–24 min)

The conventional procedure requires the operator to move to the control room, query each system, and communicate the response to the commercial department. Tasks and average times are summarized below:

1. Displacement to control room: 3–4 min (120 m, PPE removal);
2. Access and authentication in ERP: 2–3 min;
3. Availability query in MES: 3–5 min;
4. Equipment status verification in SCADA: 2–3 min;
5. Inventory and additives query in database and sensors: 1–2 min;
6. Mental integration of information and phone response: 2–3 min;
7. Return to curing area and task resumption: 3–4 min.

Estimated total time: 16–24 min. During this lapse, the curing process remains unsupervised, increasing the risk of thermal deviations or setting defects.

Method with Conversational AI (17.9 s)

In the same scenario, the operator activated the voice assistant without moving from his position:

“Assistant, can we produce 45 cubic meters of C25/30 concrete tomorrow at 7 in the morning? Verify plant availability, materials, and scheduling.”

The system processed the request with a total latency of 17.9 s, broken down as follows:

Response generated by the system: “The query is being processed. Verifying Plant 2 availability… The system indicates available capacity from 07:00. Cement level at 87%, aggregates at 92%, sufficient for 45 m^3^. C25/30 additive stock confirmed. There is a scheduled order from 06:00 to 06:45. Production viable.”

The operator maintained curing supervision without interruptions, obtaining a comprehensive synthesis of five industrial levels in a single voice interaction.

#### 3.5.3. Quantified Impact

Deployment of the conversational system reduced total response time from 960 to 1440 s (16–24 min) to only 17.9 s, with an operational improvement of 54–80× faster. Additionally, it completely eliminated physical displacement and interruption of critical tasks, while decreasing the risk of human error in interpreting or combining data from multiple systems.

Table 3 summarizes the quantitative comparison between both methods.

In terms of productivity, estimated savings equivalent to 1.3–2.0 man-hours per shift, considering an average of 5 such queries per workday, representing a potential increase of 7–9% in direct operational efficiency in plant supervision and coordination tasks.

#### 3.5.4. Latency Component Analysis

A detailed analysis of temporal breakdown (Table 4) reveals that:

- Model inference (analysis + synthesis): 9.9 s (55.3% of total time);
- Voice synthesis: 3.6 s (20.1% of total time);
- Data retrieval (5 protocols): 45 ms (0.25% of total time);
- Voice transcription: 1.2 s (6.7% of total time);
- Other processes: 3.2 s (17.9% of total time).

This analysis confirms findings from Experiment 3: industrial communication latency (45 ms cumulative for 5 accesses) represents less than 0.3% of total response time. The dominant component is language model inference (55.3%), followed by voice synthesis (20.1%).

#### 3.5.5. Qualitative Operational Impact

Beyond quantitative metrics, the case study evidences several qualitative operational benefits:

Operational continuity: The operator did not interrupt curing process supervision, maintaining visual vigilance and response capacity to deviations. In the traditional method, the 16–24 min displacement represents a risk window where thermal problems or setting issues could go unnoticed.

Cognitive load reduction: The system automatically integrated information from five heterogeneous systems, eliminating the need for the operator to remember data locations, query syntax, or authentication procedures. This cognitive load reduction is especially valuable in high operational pressure situations.

Automatic traceability: Query and response were automatically recorded in the log system (conversacion.json), providing complete decision traceability without additional operator effort. In the traditional method, this documentation would require manual entry in management systems.

Access democratization: Although the case operator had 18 months of experience, the query could have been executed with identical efficacy by an operator without prior technical training, by eliminating the need-to-know system topology, communication protocols, or physical interface location.

#### 3.5.6. Case Synthesis

The Frumecar case study shows that industrial conversational AI not only drastically accelerates information access in distributed environments (54–80× faster), but preserves operational continuity, reduces cognitive load, and democratizes multilevel system control through natural language interaction.

The response time of 17.9 s, although superior to the ideal for fluid conversation (<2 s), is perfectly viable in industrial contexts where traditional alternatives require 16–24 min. The reduction of two orders of magnitude in response time transforms operations that previously caused prolonged interruptions into practically instantaneous queries from an operational perspective.

This case empirically validates that the proposed system reaches the technical maturity necessary for industrial deployment under real conditions, providing immediate return on investment through measurable operational time savings and qualitative improvements in supervision continuity and operational risk reduction.

## 4. Discussion

### 4.1. Interpretation of Main Findings

Experimental results confirm that LLMs can execute efficiently on edge hardware [13,14,20,21], achieving response times compatible with industrial conversational applications [11,12]. Average latencies of 14.19 s for simple queries, 16.45 s for moderate queries, and 23.24 s for complex multilevel queries are situated in a viable operating range for industrial environments where traditional queries require between 15 and 30 min of manual navigation across multiple systems [1,2].

Studies in human factors demonstrate that response times below 2 s are perceived as instantaneous in conversational systems, while latencies below 30 s maintain user attention without significant loss of interactive continuity [32,33]. Our results, with average values between 14 and 23 s, empirically validate that a system based on local inference can sustain acceptable natural interaction, although not optimal, providing a substantial operational improvement of 26–77× faster compared to conventional manual query methods.

The difference between obtained times (14–23 s) and ideal times for fluid interaction (<2 s) is primarily attributed to three factors: (1) computational limitations inherent to a 4-core ARM processor without GPU acceleration, (2) the Q4_0 quantization process which, although drastically reducing memory requirements, introduces inference speed penalties on the order of 15–25% [19], and (3) the complexity of multilevel queries requiring contextual reasoning over heterogeneous data.

Quantization of the Mistral-7B model to GGUF Q4_0 format [17,18,19] reduced its size from 14 GB (FP16) to 3.82 GB, with a 68.8% decrease in memory requirements, enabling complete execution on a Raspberry Pi 5 (8 GB RAM) [22,23]. The 100% success rate observed in reliability tests confirms that cognitive degradation induced by quantization is marginal for language comprehension and generation tasks in structured industrial contexts, consistent with recent studies reporting that models reduced to 4 bits retain more than 95% of their performance in semantic comprehension tasks [19].

Quantization trade-offs. The decision to employ Q4_0 quantization represents a fundamental engineering compromise between computational feasibility and model fidelity. By reducing the Mistral-7B model from 14 GB (FP16 precision) to 3.82 GB, quantization enabled complete execution within the 8 GB memory envelope of the Raspberry Pi 5, but necessarily introduced precision degradation through weight approximation. This 72.7% reduction in memory footprint comes at the cost of representing model parameters with 4-bit integers rather than 16-bit floating-point values, potentially affecting the model’s capacity for nuanced semantic distinctions.

Recent quantization literature indicates that 4-bit schemes retain over 95% of full-precision performance in semantic comprehension tasks [19], a finding consistent with our empirical observations: across 39 industrial queries spanning three complexity levels, the system achieved 100% functional success without observable hallucinations or semantic failures. This suggests that the linguistic and reasoning demands of industrial queries—which typically involve concrete entities, structured data retrieval, and domain-constrained vocabulary—fall comfortably within the preserved capability range of quantized models. However, edge cases involving highly ambiguous phrasing, specialized technical jargon outside the training distribution, or queries requiring subtle contextual interpretation may expose the precision limitations introduced by quantization. Extended deployment across diverse operator populations and broader query patterns would be necessary to comprehensively map these boundaries and determine whether occasional precision failures emerge under real-world operational diversity.

In thermal terms, the average temperature of 69.3 °C (peak 79.6 °C) maintains a reduced operating margin of 5.4 °C below the BCM2712 SoC throttling threshold (85 °C), which represents a significant limitation [29,30,31]. This margin is critical, given that industrial environments present thermal variations of ±5–10 °C due to ambient conditions, machinery proximity, or seasonality. The total absence of frequency reduction events during the 30 min experiment confirms that, under evaluated conditions (ambient temperature of 24 °C), the active cooling system employed is sufficient. However, in environments with temperatures above 30 °C—common in industrial plants during summer—there is significant risk of thermal throttling, which would degrade performance by 20–40% according to literature [30,31].

Industrial communication latencies, with an average of 8.93 ms across 10,163 readings, represent less than 0.3% of total response time, confirming that language model inference is the dominant component of total latency, not data retrieval [4,5,6,7,28]. This finding validates the architectural strategy of maintaining enterprise data (ERP/MES) in their native systems, accessing on demand without local replication, which optimizes the use of limited storage on edge devices [13,14].

The case study documented in Section 3.5 exemplifies these findings in a real operating scenario: a multilevel production viability verification query, requiring integration of data from all five levels of the automation pyramid, was completed in 17.9 s, achieving an operational improvement of 54–80× compared to the traditional manual method (16–24 min). This result confirms that, although response times are not optimal for ideal fluid conversation, they represent a transformative improvement in real industrial contexts where conventional alternatives involve physical displacements, navigation across multiple interfaces, and interruption times measured in tens of minutes.

Overall, findings demonstrate that the combination of precision quantization, active thermal optimization, and low communication latency enables autonomous execution of language models on edge devices, enabling their practical use in manufacturing environments, although with thermal and performance considerations that must be addressed in large-scale deployments.

### 4.2. Democratization of Access to Industrial Information

The result of greatest practical impact is the drastic reduction in multilevel information access time, from 15 to 30 min (traditional manual methods) to 14–23 s (conversational system), representing an efficiency improvement of 26–77×. This acceleration eliminates prolonged operational interruptions, allows operators to maintain continuity in their physical supervision tasks, and significantly reduces the cognitive load associated with navigation across multiple heterogeneous interfaces.

From a socio-technical perspective, the proposed system redefines training processes and knowledge transfer in industrial environments [1,2]. Traditionally, training to achieve functional competency in concrete plants requires between 6 and 18 months of continuous training [3]. The use of natural language conversational interfaces [10,11,12] eliminates dependence on implicit technical knowledge, by abstracting system topology, communication protocols, and proprietary terminology. The operator does not need to know the data hierarchy or system syntax, only to express their need semantically.

This transformation has profound implications: conversational systems can accelerate the operational learning curve by up to 20–50×, reducing dependence on specialized tutors and productivity loss associated with progressive training. Moreover, this technology expands labor inclusion, by reducing technical barriers for workers with limited training, temporary personnel, or generations with lower digital literacy, thus contributing to the resilience and social sustainability objectives of Industry 5.0 [1].

However, it is important to recognize that obtained response times (14–23 s), although substantially better than manual methods, still present perceptible latencies that could affect the fluidity of prolonged conversational interactions. For queries requiring multiple consecutive exchanges, cumulative time could generate cognitive friction. Future optimizations through specialized hardware (e.g., Google Coral TPU, Intel Neural Compute Stick) or smaller models (3B–5B parameters) could reduce these times to the 5–10 s range, significantly improving user experience without compromising cognitive capabilities [21,29].

### 4.3. Communication Efficiency with Heterogeneous Industrial Protocols

Latency analysis demonstrates that heterogeneous data retrieval [4,5,6,7,28] does not constitute a bottleneck for edge inference. The average latency of 8.93 ms, with a maximum of 31.12 ms under network load conditions, confirms stable network behavior without congestion or perceptible jitter, ensuring temporal predictability, a critical requirement for operator trust in industrial conversational systems.

The detailed breakdown of the Frumecar case (Table 4, Section 3.5) empirically confirms this observation: of the total 17.9 s of a complex multilevel query, only 45 ms (0.25%) corresponded to cumulative latencies of five heterogeneous protocols distributed across the five levels of the automation pyramid (OPC UA for PLC, MQTT for field sensors, and REST API for MES and ERP). In contrast, language model inference consumed 9.9 s (55.3%) and voice synthesis 3.6 s (20.1%). This result validates that optimization effort should focus primarily on accelerating model inference through specialized hardware or optimized models, not on industrial communication infrastructure.

From an architectural perspective, these results validate that the on-demand remote access strategy to MES/ERPs is more sustainable than architectures that locally replicate enterprise databases, which suffer from synchronization problems, eventual consistency, and excessive storage consumption in edge environments with limited resources [13,14]. The maximum cumulative latency observed for a real multilevel query (45 ms for five protocols) is negligible compared to model inference times (14–23 s), confirming that future optimization should focus on inference acceleration, not communication.

The proposed architecture also facilitates horizontal scalability: new automation levels or enterprise systems can be integrated through addition of new protocol clients (e.g., Modbus, BACnet) without modifying the model inference core. This modularity is essential for heterogeneous industrial environments where integration of legacy systems and emerging technologies is an operational constant.

### 4.4. Comparison with Cloud-Based Solutions

The proposed local system presents significant advantages over cloud inference solutions [8] in terms of latency, autonomy, privacy, and operational cost. Commercial services such as GPT-4 [8], Claude, or Gemini [15] present typical network latencies of 200–800 ms per conversational exchange, which accumulated in multilevel queries can raise total response times to 15–25 s, comparable or superior to those of the evaluated local system.

Additionally, industrial environments typically operate with strict OT/IT segmentation and cybersecurity policies [16] that restrict Internet access from operational networks, making cloud solutions vulnerable to connectivity failures, corporate blocks, or denial-of-service attacks. The local inference approach guarantees 24/7 operational autonomy, even during external network interruptions, eliminating critical dependencies on third-party infrastructure.

Industrial data privacy is a critical differentiator: local inference ensures that no sensitive data—production scheduling, inventory levels, process parameters, logistical information—leaves the facilities. This eliminates corporate exposure risks, complies with data protection regulations (GDPR, CCPA), and ensures security policy compliance without requiring end-to-end encryption infrastructure or costly external audits.

### 4.5. Study Limitations

Although results are conclusive, several methodological limitations must be considered. Validation was performed for three weeks at a single industrial installation (Frumecar, Murcia, Spain) in the precast concrete sector, so results do not capture cross-sector variability or long-term behavior. Extended trials (6–12 months) across diverse sectors would be necessary to evaluate seasonal thermal variations, preventive maintenance cycles, and system evolution.

The system was tested exclusively with native peninsular Spanish speakers under moderate ambient noise conditions (<65 dB) and controlled temperature (24 ± 1 °C). Voice recognition accuracy may degrade 10–25% with Latin American Spanish variants or marked regional accents, and environments with temperatures above 30 °C could induce thermal throttling that would degrade performance by 20–40%. Additionally, the set of 15 evaluated queries, although representative of daily operations, does not cover extreme cases involving complex temporal constraints, deep causal reasoning, or predictive diagnosis.

From a methodological perspective, standardized usability instruments (SUS, NASA-TLX) were not applied, limiting characterization of cognitive load and user acceptance. The system operates exclusively in query mode (read-only) without capability to execute control commands, and while hallucinations are mitigated through low temperature (T = 0.3) and direct access to real data sources, extended operational periods would require systematic monitoring. Finally, the experimental validation was conducted exclusively on Raspberry Pi 5 hardware, and the 1024-token context window limits sustained conversations to 3–5 exchanges.

#### 4.5.1. Safety and Control Barriers

The system operates exclusively in query mode (read-only) without capability to execute control commands or modify actuators, eliminating risks of incorrect physical actions derived from LLM outputs. Hallucinations are mitigated through: (1) low temperature (T = 0.3) ensuring deterministic responses, and (2) direct access to real industrial data sources (OPC UA, MQTT, REST API) avoiding speculative generation. The multi-layer architecture (Figure 1) provides separation of responsibilities where the LLM functions solely as a semantic interpreter, not as an executor of critical industrial actions. This design choice prioritizes operational safety in accordance with industrial automation standards.

#### 4.5.2. Hardware Platform Dependency and Model Limitations

The experimental validation was conducted exclusively on Raspberry Pi 5 hardware (ARM Cortex-A76, 8 GB RAM, Raspberry Pi Foundation, Cambridge, UK), which constrains generalizability of performance metrics to this specific platform. Alternative edge devices—such as NVIDIA Jetson or Intel NUC systems—present different computational profiles and thermal characteristics that would alter system performance. The tight thermal margin observed (5.4 °C below throttling threshold) particularly limits deployment in fanless or high-temperature industrial environments.

The Mistral-7B language model, despite mitigation strategies (temperature T = 0.3, direct data access, read-only mode), remains susceptible to semantic misinterpretations of ambiguous queries or generation of plausible but incorrect responses. The Q4_0 quantization, while enabling edge execution, introduces precision loss that may degrade nuanced language understanding. Although the 30 min reliability test showed no hallucinations across 39 queries, extended operational periods would require systematic monitoring and user feedback mechanisms.

Scalability presents constraints across multiple dimensions: the 1024-token context window limits sustained conversations to 3–5 exchanges, linguistic scope is restricted to Spanish, and the current architecture does not accommodate concurrent multi-user scenarios without additional queuing and load balancing mechanisms.

### 4.6. Future Directions

Results open several strategic development lines. In terms of performance optimization, integration of specialized neural processing units such as Google Coral TPU or Intel Neural Compute Stick could reduce inference times by 3–5×, achieving responses in the 3–8 s range. Alternatively, smaller specialized models (3B–5B parameters) adapted through parameter-efficient fine-tuning techniques (LoRA, PEFT) could offer an optimal balance between performance and latency for structured industrial queries.

Multimodal extensions represent another promising direction. Incorporation of computer vision models would enable queries combining visual perception with linguistic reasoning for assisted diagnosis and quality control, while gesture recognition could provide alternative input modalities for high-noise environments where voice is not viable. Additionally, implementing explicit feedback mechanisms would enable iterative model adjustment through reinforcement learning from human feedback (RLHF), aligning system behavior with operator preferences.

From an architectural perspective, extending the system toward explanatory failure diagnosis through integration of causal graphs and temporal event analysis would enable response to queries requiring root cause identification. Multi-agent architectures that decompose the monolithic reasoning model into specialized agents aligned with the automation pyramid’s hierarchical structure could reduce individual model complexity and enable parallel processing. Finally, federated learning approaches could enable collaborative model improvement across geographically distributed plant deployments while preserving data privacy.

#### Scalability to Larger Models and Multi-Agent Architectures

The current implementation employs Mistral-7B as a deliberate compromise between linguistic capability and edge hardware constraints, but alternative architectural strategies could reshape this trade-off space in multiple directions. Smaller specialized models in the 3B–5B parameter range, adapted to industrial domains through parameter-efficient fine-tuning techniques [20], represent one promising avenue. Such models could potentially reduce response latencies from the current 14–23 s range to 5–10 s while maintaining adequate performance for structured industrial queries, particularly if fine-tuning incorporates domain-specific corpora that emphasize the technical vocabulary and query patterns characteristic of manufacturing environments.

Conversely, scaling upward to larger models (13B–70B parameters) would exceed the memory capacity of single-board computers, necessitating distributed inference architectures. In such configurations, computationally intensive transformer layers could be offloaded to edge servers with greater resources, while latency-critical components (voice processing, protocol communication) remain local. This hybrid topology would preserve the privacy and autonomy advantages of edge deployment while selectively accessing additional computational capacity when query complexity demands it.

Beyond vertical scaling, multi-agent architectures offer an alternative paradigm that decomposes the monolithic reasoning model into specialized agents aligned with the automation pyramid’s hierarchical structure [26]. A field-agent specialized in sensor data interpretation, a control-agent optimized for PLC/SCADA queries, and a planning-agent trained on MES/ERP semantics could operate in parallel, coordinated by a meta-agent responsible for query routing and response synthesis. This decomposition would reduce individual model complexity, enable parallel processing across automation levels, and facilitate incremental updates to domain-specific agents without requiring complete system retraining. Furthermore, federated learning approaches [34] could enable collaborative model improvement across geographically distributed plant deployments: each local system would contribute to collective refinement through gradient sharing or model distillation, while sensitive operational data never leaves individual facilities, preserving privacy guarantees central to the edge deployment rationale.

### 4.7. Implications for Industry 5.0

Findings directly contribute to the emerging Industry 5.0 paradigm [1], which promotes human–machine collaboration, operational resilience, and social sustainability above the techno-centric approach of Industry 4.0 [2].

Democratization of cognitive access to industrial information enables reincorporation of human judgment into the control loop, strengthening contextual decision-making and adaptability to unforeseen events. While Industry 4.0 emphasizes automation that replaces human work, Industry 5.0 proposes augmentation that enhances human capabilities, creating systems where artificial intelligence acts as a cognitive amplifier rather than replacement.

This technology expands labor inclusion by reducing technical barriers for workers with limited training, temporary personnel, older adults with lower digital literacy, or people with disabilities that hinder use of traditional graphical interfaces. Access through natural language—universal communication modality—transforms complex industrial systems into accessible interfaces for anyone with linguistic capability, regardless of their technical training.

Moreover, the local inference approach eliminates dependencies on centralized cloud infrastructure, increasing operational resilience against network interruptions, cyberattacks, or external provider failures. This autonomy is critical for strategic sectors (energy, water, health, food) where operational continuity is a national security requirement.

From a sustainability perspective, the proposed model reduces energy consumption associated with remote data centers (typically 0.3–0.5 kWh per 1000 queries in cloud solutions) by executing inference locally with a consumption of 5–8 W from the Raspberry Pi, reducing operational carbon footprint by approximately 90% compared to cloud alternatives.

Ultimately, conversational AI at the edge [13,14,20] represents a transition from automation that replaces toward augmentation that enhances human capabilities, offering an operational framework where knowledge is shared immediately, contextually, and securely. This paradigm is essential for maintaining industrial competitiveness and resilience in scenarios of high variability, specialized talent scarcity, and growing demands for customization and sustainability.

### 4.8. Implications and Contributions

The contributions of this work establish a new benchmark for running large-scale language models in industrial edge computing, demonstrating that a quantized Mistral-7B model in Q4_0 can operate entirely on a Raspberry Pi 5 with full thermal stability, 100% operational success, and no throttling events. This result breaks with the conventional notion that local LLM inference requires GPUs or dedicated infrastructure, providing empirical evidence that the combination of extreme quantization, thermal optimization, and modular architecture enables the viability of autonomous industrial assistants running on cost-effective and energy-efficient hardware.

The study also provides a rigorous characterization of multi-layer industrial latencies, demonstrating that the bottleneck in real-world environments lies not in the plant protocols (MQTT, OPC UA, REST), whose latencies remain below 10 ms, but rather in the model’s linguistic inference. This distinction, supported by over 10,000 measurements and comprehensive statistical analyses, validates a key architectural principle: maintaining ERP/MES/SCADA systems in their native environments and accessing them on demand is not only possible, but optimal for conversational systems based on edge AI.

Finally, the case study demonstrates an unprecedented operational impact, reducing multi-source validations from 16 to 24 min to just 17.9 s (a 54–80× improvement), eliminating physical travel, cognitive load, and associated operational risks. This evidence confirms that the proposal is not only technically feasible, but also organizationally transformative, establishing a new paradigm for democratizing access to industrial data through natural language interaction, with tangible benefits in productivity, operational continuity, and automated traceability.

## 5. Conclusions

This study demonstrates the technical and operational feasibility of deploying conversational artificial intelligence based on large language models on low-cost edge hardware, enabling cognitive access to information distributed across all five levels of the industrial automation pyramid. The experimental validation at Frumecar S.L. confirms that a quantized model can execute entirely on a Raspberry Pi-class device with response times acceptable for industrial operations, transforming previously disruptive information-seeking tasks into brief interactions that preserve operational continuity.

Main contributions. First, we present an end-to-end industrial cognitive architecture that integrates speech interaction, heterogeneous industrial protocols (OPC UA, MQTT, REST APIs), and contextual reasoning in Spanish through a unified conversational interface. Second, we demonstrate the computational and thermal viability of quantized language models on affordable edge hardware, operating reliably without throttling under controlled ambient conditions while identifying constraints for high-temperature environments. Third, we show that communication with industrial data sources contributes only marginally to overall latency, validating the architectural decision to access enterprise and operational data on demand rather than through local replication.

Operational impact. The deployment demonstrates industrial-grade robustness with complete autonomy from external connectivity and cloud services. Fully local inference ensures that sensitive operational data remain within the plant, simplifying compliance with OT/IT segmentation policies and eliminating recurring subscription costs. By enabling natural language access to distributed industrial information, the system acts as a resilience vector that strengthens operator decision-making capacity rather than replacing human expertise.

Limitations. This work focuses on a single industrial installation in the precast concrete sector with native peninsular Spanish speakers, limiting generalizability across sectors and dialectal diversity. The evaluation emphasizes objective operational metrics; subjective dimensions such as cognitive load and perceived usability require systematic assessment in future studies.

Future directions. Promising research avenues include multi-site longitudinal deployments across diverse industrial sectors, dialectal evaluations with speakers from different Spanish-speaking regions, structured usability studies using established instruments (SUS, NASA-TLX), multimodal extensions combining voice with gestures and computer vision, and advanced reasoning capabilities for causal analysis based on alarm correlations and subsystem dependencies.

In summary, the convergence of quantized language models, affordable edge hardware, and standardized industrial protocols has reached sufficient maturity to transform human–system interaction in manufacturing environments. The Frumecar deployment demonstrates that such solutions can operate reliably in production while ensuring data privacy, operational autonomy, and rapid return on investment, positioning conversational AI as a practical pathway toward cognitive democratization and resilient Industry 5.0 manufacturing.

## Figures and Tables

**Table 1:** Response times (seconds) by query complexity.

Complexity	N	Mean	SD	Median	Q1	Q3	Min	Max
Simple	5	14.19	7.56	14.80	7.71	20.76	5.39	22.28
Moderate	5	16.45	6.40	15.64	11.73	19.84	9.55	25.49
Complex	5	23.24	6.59	19.97	18.84	25.84	17.92	33.65

**Table 2:** Operational reliability metrics during 30 min of autonomous operation.

Metric	Value
Total executed queries	39
Successful completions	39
Partial failures (recovered)	0
Critical failures (crash)	0
Success rate	100%
Uptime	100%
Average memory usage	5.2 GB
Peak memory usage	5.8 GB
Average CPU temperature	69.3 °C
Peak CPU temperature	79.6 °C

**Table 3:** Operational efficiency comparison for production viability verification.

Metric	Traditional Method	Conversational AI	Improvement
Total time	16–24 min	17.9 s	54–80×
Physical displacement	240 m	0 m	100% elim.
Manually consulted systems	4	0 (automated)	100% elim.
Supervision interruption time	16–24 min	0 s	100% elim.
Data transcription error risk	Moderate	Minimal	N/A

**Table 4:** Total latency breakdown.

Stage	Duration
Voice transcription	1.2 s
Intent analysis and query planning (Mistral-7B)	2.7 s
Data retrieval:	
- Plant 2 status (OPC UA—PLC)	9 ms
- Cement silo level (MQTT)	9 ms
- Aggregate levels (MQTT)	9 ms
- ERP additive inventory (REST API)	9 ms
Response synthesis (Mistral-7B)	7.2 s
Voice synthesis and emission	3.6 s
Total	17.9 s

## Data Availability

The original contributions presented in this study are included in the article material.
